# Sustained Self-Regulation of Energy Intake: Initial Hunger Improves Insulin Sensitivity

**DOI:** 10.1155/2010/286952

**Published:** 2010-06-22

**Authors:** Mario Ciampolini, David Lovell-Smith, Riccardo Bianchi, Boudewijn de Pont, Massimiliano Sifone, Martine van Weeren, Willem de Hahn, Lorenzo Borselli, Angelo Pietrobelli

**Affiliations:** ^1^Unit of Preventive Gastroenterology, Department of Paediatrics, Università di Firenze, 50132 Florence, Italy; ^2^Department of General Practice and Primary Health Care, University of Auckland, Auckland, New Zealand; ^3^Department of Physiology and Pharmacology, Robert F. Furchgott Center for Neural and Behavioral Sciences, State University of New York Downstate Medical Center, Brooklyn, NY, USA; ^4^AMC, 1100 DD Amsterdam, The Netherlands; ^5^Department of Statistics, Università di Firenze, Florence, Italy; ^6^Paediatric Unit, Università di Verona, Verona, Italy

## Abstract

*Background*. Excessive energy intake has been implicated in diabetes, hypertension, coronary artery disease, and obesity. Dietary restraint has been unsuccessful as a method for the self-regulation of eating. Recognition of initial hunger (IH) is easily learned, can be validated by associated blood glucose (BG) concentration, and may improve insulin sensitivity. *Objective*. To investigate whether the initial hunger meal pattern (IHMP) is associated with improved insulin sensitivity over a 5-month period. *Methods*. Subjects were trained to recognize and validate sensations of IH, then adjust food intake so that initial hunger was present pre-meal at each meal time (IHMP). The purpose was to provide meal-by-meal subjective feedback for self-regulation of food intake. In a randomised trial, we measured blood glucose and calculated insulin sensitivity in 89 trained adults and 31 not-trained controls, before training in the IHMP and 5 months after training. *Results*. In trained subjects, significant decreases were found in insulin sensitivity index, insulin and BG peaks, glycated haemoglobin, mean pre-meal BG, standard deviation of diary BG (BG as recorded by subjects' 7-day diary), energy intake, BMI, and body weight when compared to control subjects. *Conclusion*. The IHMP improved insulin sensitivity and other cardiovascular risk factors over a 5-month period.

## 1. Introduction

In industrialised countries, most people regulate their energy expenditure poorly. Individual energy expenditure may differ up to 20-fold between resting conditions and high physical activity, but such differences have until now been weakly correlated to energy intake at subsequent meals [1]. Frequent episodes of positive energy balance can lead to insulin resistance, overweight, obesity, diabetes, and heart disease [1, 2]. Dietary regimes that attempt to restrain eating have been only marginally successful [3, 4] and the feasibility of self-regulation of energy intake regimes has been questioned [5]. A key reason for this lack of success may be that most dietary methods rely on weekly or monthly measurements of weight. These measurements provide no immediate feedback to dieters, who usually ingest food at least three times daily.

The body's own physiological signaling system is hunger. Blood glucose concentration (BG) is a reliable index of energy availability to body cells [6–8]. It seems reasonable to assume that BG slowly declines in the absence of food intake during the day until hunger emerges to trigger eating behaviour [9, 10]. Previous studies suggested that waiting for hunger before eating is associated with a significant decrease in energy intake [11–15].

Subjects can be trained to predict when BG is low by attending to their subjective experience of hunger [16]. Thus low blood glucose (LBG) can be regarded as a biochemical marker for hunger. The first intimations of hunger we term Initial Hunger (IH), to differentiate it from the uncomfortable symptoms that occur when hunger is prolonged. IH is not a reflex conditioned by external events such as time or social circumstances [17]. For example, IH is not conditioned by meal times since it arises unexpectedly (outside meal times) if energy content of the previous meal was not planned to cover the intermeal interval [16]. The Initial Hunger Meal Pattern (IHMP) is a pattern of eating such that IH is present before most meals. We reasoned that the IHMP should predict closely regulated BG concentration with associated improvements in metabolic biomarkers.

In this study, we tested the hypothesis that the IHMP is associated with improvements in metabolic biomarkers, in particular insulin sensitivity.

## 2. Methods

### 2.1. Participants

#### 2.1.1. Eligibility Criteria

**Figure 1:**
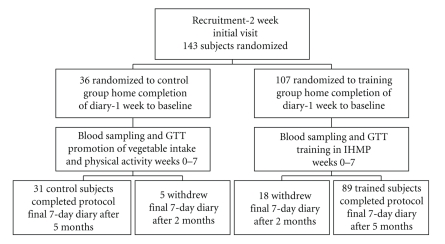
*Consort flow chart and investigation design*. Randomized controlled 5-month clinical investigation to study the metabolic effects of the IHMP.

The Paediatric Gastroenterology Unit of Florence University recruited 143 subjects to this study from 1996 to 2000. This unit diagnoses and treats celiac disease in children and adults. Aged 18 to 60 years, subjects suffered from symptoms of functional bowel disorders such as dyspepsia, abdominal pain, and diarrhoea (Figure 1) [18, 19]. They showed no morphological, physical, or biochemical signs of organic disease [11, 18, 19]. Subjects with impaired glucose tolerance (fasting plasma-glucose >115 mg/dL (6.4 mmol/L)), and noninsulin dependent diabetes mellitus (NIDDM), celiac, liver, heart, brain, thyroid, and kidney diseases were excluded from this study (Figure 1). Written informed consent was obtained from all subjects. The local Hospital Ethics Committee approved the study in compliance with the Helsinki Declaration.

#### 2.1.2. Setting

The trained group continued their regular work or recreational activities under tutorial assistance for seven weeks and maintained the IHMP for a further three months independently (Figure 1).

### 2.2. The Intervention

Subjects were trained in the IHMP, first by identifying IH, which was guided by consistency in subjective sensations and the association of these sensations with BG measurement. During training, subjects measured capillary blood by portable glucometer (Glucocard Memory; Menarini Diagnostics; Florence, Italy) in the 15 min before a meal. Accuracy of measurements by the glucometer was validated against periodic measurements by hospital autoanalyzer. Seven-day home diaries reported BG measurements and presence or absence of IH before the three main meal times. Also recorded in the diary were energy and vegetable intake, hours in bed, and hours spent during physical and outdoor activities (weekly mean and SD). Subjects were advised that BG measurements after taking small quantities of food (even a few grams), after changes in ambient temperature, after physical activity such as walking or cycling, and when under psychological stress would be misleading since we had previously found that BG and IH do not correlate well under these conditions [16].

Subjects reported IH as gastric pangs, sensations of emptiness and hollowness, and mental or physical weakness [16]. IH was cultivated pre-meal by adjusting composition, portion size, or timing of food intake. After a few days of trial and error, and sometimes irregular meal times, subjects were able to arrange their food intake so that IH appeared before the usual three meal times per day with an average error of half-an-hour in 80% of instances [15, 16, 20–23]. Training ended after the first 7 weeks, to be resumed only at the end of the investigation. Thus, after the first 7 weeks, subjects relied upon the identified subjective sensation (IH) alone, as the signal to begin a meal. Control subjects (*N* = 31) were given the same information on food energy content and were recommended vegetable intake and physical activity per day as were the trained subjects (weeks 0–7, Figure 1).

120 subjects who completed the study were assessed for blood parameters at baseline (before training), after the first 7 weeks of training, and at the end of the investigation after a further three months (total duration of the investigation: 5 months). During the glucose tolerance test, after a 12-hour overnight fast, all subjects were given a 75 g-oral glucose load. Venous blood samples were taken immediately before glucose was administered, and 30, 60, 90, 120, and 180 min thereafter to determine plasma glucose and serum insulin. Serum insulin was measured with the IMx insulin assay (Abbott Lab. Diagn. Div. USA) [24]. From the glucose tolerance test (GTT), we calculated the area under the curve (AUC), the index of whole-body insulin sensitivity (10,000/square root of [fasting glucose × fasting insulin] × [mean glucose × mean insulin during GTT]) [25], and the insulinogenic index of beta cell function (ratio of the increment of plasma insulin to that of plasma glucose 30 min after glucose loading) [26].

### 2.3. Outcomes

#### 2.3.1. Primary Endpoint

The primary endpoint was the change in insulin sensitivity [25] from baseline at 5 months in trained subjects compared to controls.

#### 2.3.2. Secondary Endpoints

Analyses were also performed on beta cell function [26], BG AUC, GTT measurements of BG and insulin concentrations, and mean pre-meal BG and HbA1c values [27] as well as energy intake, BMI, body weight and arm and leg skinfold thickness.

### 2.4. Sample Size

Previous work in similar patients found that the insulin sensitivity index in the intervention group was greater by 3 than that in the control group, with a standard deviation (SD) of 3.0 [23]. Based on these figures, our sample size calculations suggested that we needed a minimum of 14 subjects in each comparison group to detect a similar difference in group means, with a power of 80% and a 1 sided alpha of 0.05.

### 2.5. Randomization

A list was divided into blocks of 1 to 4 places, and the blocks were randomly assigned using Armitage even and odds random numbers on a 3  :  1 ratio to either training or control groups. A dietician kept the list and subsequently assigned each recruited subject to the first empty list place. Control or training destination was revealed after the first visit (Figure 1).

### 2.6. Statistical Methods

**Figure 2:**
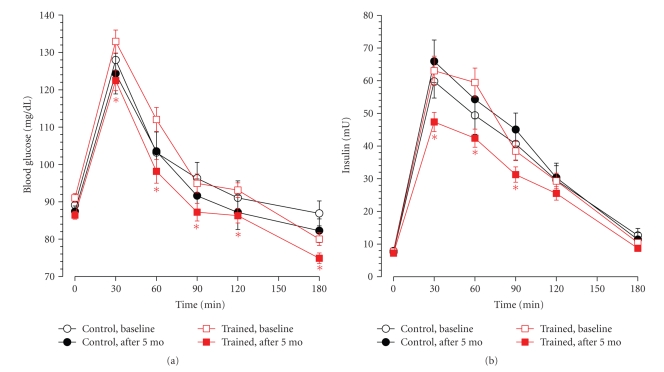
*Blood glucose and plasma insulin concentrations during GTT in control and trained subjects at the beginning and at the end of the study*. Blood glucose (a) and insulin (b) mean levels in control (black circles) and trained (red squares) subjects at baseline (open symbols) and after 5 months (closed symbols). Vertical bars are standard errors. Asterisks indicate significant decrease of blood glucose (a) and insulin (b) in the trained subjects after training compared to their respective baseline values (*P* < .01). In contrast, no decrease between baseline values and those at the end of the study was observed in control subjects. The insulin decrease in trained subjects at 60 and 90 min also differed significantly from the control group (*P* < .01 and <.05, resp.).

Values are expressed as means ± SD, except in Figure 2, where the Standard Error is shown. Logistic regression analysis was used to investigate the association of training with BG mean, Hb1c, insulin and BG AUCs, intakes and anthropometric measures (trained versus untrained control groups) for significance of multiple results [28]. The significance of difference and correlation was set at *P* < .05 in these analyses. Yates test and two-tailed Student's *t*-test on paired or unpaired samples according to data requirements were used to analyse the significance of difference and two-tailed Student's *t*-test for correlation. The significance was set at *P* < .05 for single measurements and at *P* < .025 for the GTT insulin and BG AUCs [29]. Custom-made software was used to tabulate data for statistical analyses. Microsoft Excel (Microsoft Corp., USA) and SAS 8 (SAS Institute Inc., Cary, NC, USA) were used for data presentation and for statistical analyses.

A training effect and correlations between the two body size parameters (weight and BMI), the two energy-balance parameters (arm and skinfold thickness), the four metabolic indexes (mean BG and HbA1c values, and BG and insulin AUCs), and three intake factors (energy, fruit, and vegetable) were longitudinally investigated (i.e., on post minus predifferences) by simple, linear correlation and regression analyses in all of the 120 subjects completing the study (Figure 1). Results were validated by chi square test-collinearity diagnostics-residual analysis.

## 3. Results

Figure 1 shows the flow chart of participants through each phase of the study. Data were eventually collected from 120 subjects who completed the study (60 females and 60 males, 89 trained subjects and 31 control subjects).

### 3.1. Losses and Exclusions

#### 3.1.1. Protocol Deviations

In this study the protocol was to follow the IHMP. We do not have data on the extent to which IH was present pre-meal for each meal, that is, we do not know how closely each subject adhered to the IHMP. Achieving the IHMP appeared to be difficult for 12 subjects who had high pretraining mean BG concentrations (e.g., around 100 mg/dL) or participated in heavy manual labour, especially in cold conditions. Although some subjects may not have been faithful to the IHMP for all meals, we have included all those who completed the study in the final analysis, since it was our intention to treat them [30, 31].

#### 3.1.2. Dropouts

Twenty-three subjects (18 trained and 5 control) did not complete the study (dropouts). All were contacted by telephone. Their given reasons were that they “required no further training” or had “busy schedules.” To ascertain whether these biases could have affected the generalisability of the study's conclusions, we performed a sensitivity analysis using baseline and 7-week data from all 23 dropouts. The 18 trained dropouts significantly decreased mean BG (from 83.3 ± 5.9 mg/dL to 78.9 ± 5.4 mg/dL; *P* = .005), energy intake (from 1651 ± 451 to 1124 ± 401; *P* = .0001), BMI (from 23.7 ± 3.4 to 22.9 ± 3.2; *P* = .04), and arm skinfold thickness (from 20.5 ± 8.5 to 18.5 ± 8.8; *P* = .03). The 5 control dropout subjects showed no change in these assessments.

### 3.2. Baseline Demographics

Since no significant gender difference in baseline mean BG concentrations was observed in the control group (females: 82.3 ± 8.0 mg/dL; *N* = 14; and males: 87.5 ± 7.6 mg/dL; *N* = 17; Student's *t*-test for unpaired data: *P* = .075) and in the training group (females: 84.3 ± 8.7 mg/dL; *N* = 46; and males: 87.5 ± 10.6 mg/dL; *N* = 43; *P* = .115), the measurements from both genders were pooled in each group (Figure 1). Baseline BG means of the control subjects (85.2 ± 8.1 mg/dL; *N* = 31) did not differ from those of the training subjects (85.9 ± 9.7 mg/dL; *N* = 89; *P* = .733).

Baseline values of mean age, school education years, body weight, BMI, arm and leg skinfold thickness, and blood values did not significantly differ between control and trained groups (Tables 1 and 2).

### 3.3. Outcomes

Significant decreases among trained subjects compared to controls were found in insulin sensitivity index, insulin and BG peaks, insulin at 60 minutes and 90 minutes during GTT, glycated haemoglobin, mean pre-meal BG, BG diary standard deviation (SD), energy intake, BMI, body weight, arm and leg skinfold thickness.

Index of beta cell function changed from 1.0 ± 0.8 to 1.1 ± 1.1 in trained subjects and from 1.0 ± 1.0 to 0.7 ± 0.6 in control subjects. These changes were not significant. Insulin and BG AUCs in the trained group significantly decreased in the pre/postcomparison but the decreases were not significantly different from those of the control subjects. 

A significant decrease of preprandial BG mean values achieved during training was maintained three months after the training period ceased (baseline: 85.6 ± 9.5 mg/dL; after 5 months: 79.4 ± 6.5 mg/dL; *N* = 89; Student's *t*-test for paired data: *P* < .0001) (Table 2). In contrast, mean preprandial BG in control subjects did not change from baseline (baseline: 85.2 ± 8.1 mg/dL; after 5 months: 85.3 ± 7.6 mg/dL; *N* = 31; *P* = .935) and the longitudinal difference from the trained group was significant (*P* < .001; Table 2). 

#### 3.3.1. Ancillary Analyses

The absolute pre/post change (increase or decrease) in 31 control subjects was 6.0 ± 4.6 mg/dL (13.2% ± 10.1% of the baseline range in mean BG in the 120 investigated subjects: 64.5 mg/dL to 109.9 mg/dL). Factors that most characterized the differences between the trained group and the control group were investigated in all 120 subjects together by a logistic regression analysis. Energy intake (*P* = .004) and HbA1c (*P* = .0001) were significantly and negatively associated with the training. Further effects associated with training were investigated by stepwise regression analysis. The training was significantly and negatively associated with BMI (*P* = .001) and with arm and leg skinfold thickness (balance during the 5 months of investigation; *P* = .005 and *P* = .015, resp.). Decrease in BMI by training was significantly associated with decreases in energy intake (*P* = .001) and insulin AUC (*P* = .001). Analysis of weight confirmed the BMI findings.

### 3.4. Adverse Events

Trained subjects reported few negative effects when adjusting their food intake and in accommodating irregular intermeal intervals in the first few days of trial and error. The reported adverse effects included a slightly depressed BG (below 60 mg/dL (3.3 mmol/l)) and weakness or abdominal pain.

## 4. Discussion

### 4.1. Limitations of the Study

The high number of dropouts is an important limitation of this study. However, from our sensitivity analysis, we conclude that the dropout subjects are unlikely to represent a significantly different population with respect to the endpoint measures of this study and that the absence of final data from these subjects is unlikely to have significantly affected the overall results.

### 4.2. Generalisability

Our findings are from subjects who attended a gastroenterology clinic over a 5-month period. Further investigation will be necessary to evaluate the effect of the IHMP in other populations and what “reminder” training might be necessary to ensure compliance with the IHMP over years.

### 4.3. Interpretation

#### 4.3.1. Synopsis of Key Findings

A seven-week training program to establish the IHMP led to significant decreases in insulin sensitivity index, insulin and BG peaks, glycated haemoglobin, mean pre-meal BG and BG diary SD. Energy intake, BMI, and body weight also significantly decreased.

#### 4.3.2. Possible Mechanisms and Explanations

IH may represent an important afferent arm of a physiological regulation mechanism that provides meal-by-meal feedback on energy need thus optimizing energy intake. The observed improved insulin sensitivity may reflect lowered energy intake resulting from the IHMP.

#### 4.3.3. Comparison with Previous Findings

Before training, mean pre-meal BG showed high intersubject variability, in agreement with other authors' findings. This variability has engendered a perception that BG has no relevance to food intake regulation [8]. The mean pre-meal BG in trained subjects decreased significantly over 5 months, whereas control subjects showed no change. We suggest, therefore, that inter-subject variability arises because in many subjects hunger (and thus LBG) is, by habit, forestalled by premature food intake leading to sustained mild hyperglycemia. That the absolute pre/post change (increase or decrease) in pre-meal BG was modest in 31 control subjects (13.2% ± 10.1% of baseline range in mean BG variation of 120 investigated subjects) supports the contention that in untrained subjects eating occurs according to long-standing habit.

#### 4.3.4. Clinical and Research Implications

We suggest the IHMP offers a viable alternative to low fat and low carbohydrate diets [32] that is safe, cost-effective, and likely to be met with greater acceptance since it does not involve energy deprivation.

The ramifications of improved insulin sensitivity extend well beyond glucose homoeostasis [33–36]. For example, the chronic subclinical inflammation indicated by C reactive protein (CRP) is now seen as part of the insulin resistance syndrome [33, 35]. Our results could thus have implications in a variety of inflammatory conditions. Trained subjects showed a cumulative energy balance that was negative after 5 months, and the longitudinal difference was significant in comparison with control subjects. Elsewhere, we describe the effect of the IHMP on body weight in relation to baseline weight and mean BG, using a larger sample size [23].

## 5. Conclusions

Our data suggest that (i) IH provides meal-by-meal feedback allowing the conscious formation of a new eating pattern (IHMP) and sustained self-regulation of energy intake, and (ii) over a five-month period the IHMP is associated with improvement in insulin sensitivity, LBG, HbA1c, and other cardiovascular risk factors.

These findings, together with those of an associated study on weight [23], suggest that the current epidemic of insulin resistance and overweight may have its origin in noncognizance of hunger. This may owe to habitual forestalling of hunger in early life and subsequent reinforcement of this behaviour pattern. By restoring and validating hunger, the IHMP could help in the prevention and treatment of diabetes and obesity and associated disorders. This could lessen the high economic burden of health services in industrialised societies.

## Figures and Tables

**Table 1:** Group composition and effects of training on anthropometry.

	Control		Trained	
	Baseline	After 5 mo.	Baseline	After 5 mo.
Number of subjects and Gender	14 F + 17 M		46 F + 43 M	
Schooling (years)^1^	10.6 ± 3.2		12.0 ± 2.7	
Age (years)^1^	29.6 ± 8.2		32.6 ± 8.5	
BMI	22.2 ± 4.5	22.5 ± 3.7	23.0 ± 3.8	22.1 ± 3.1^∗∗∗a ∗∗∗b^
Weight (Kg)	59.6 ± 9.5	60.9 ± 8.8	64.1 ± 12.7	62.0 ± 11.3^∗∗∗a ∗∗∗b^
Arm skinfold thickness (mm)	15.2 ± 9.8	14.6 ± 7.7	16.0 ± 8.0	13.0 ± 6.1^∗∗a ∗∗∗b^
Leg skinfold thickness (mm)	20.6 ± 12.3	19.8 ± 11.0	21.6 ± 11.1	17.4 ± 8.5^∗∗a ∗∗∗b^

Values are expressed as means ± SD. ^1^Values at the beginning of the study. *Asterisks* indicate significance (Student's *t*-test: *P < .05; **P < .01; ***P < .001) of longitudinal difference versus respective control group (a), or versus baseline values of the same group (b).

**Table 2:** Effects of training on metabolic and intake parameters.

	Control		Trained	
	Baseline	After 5 mo.	Baseline	After 5 mo.
Mean pre-meal BG (mg/dL)	85.2 ± 8.1	85.3 ± 7.6	85.6 ± 9.5	79.4 ± 6.5^∗∗∗a ∗∗∗b^
BG diary SD (mg/dL)^1^	8.4 ± 3.0	9.1 ± 3.2	8.4 ± 4.4	6.1 ± 2.4^∗∗∗a ∗∗∗b^
Glycated Hb (%)	4.55 ± 0.37	4.71 ± 0.40	4.71 ± 0.4.2	4.50 ± 0.43^∗∗∗a ∗∗∗b^
Insulin AUC^2^ (mU L^−1^3 h^−1^)	211 ± 91	225 ± 111	220 ± 127	171 ± 89^∗∗∗b^
Insulin peak (mU L^−1^)	71 ± 32	74 ± 38	72 ± 46	55 ± 29^∗∗a ∗∗∗b^
Insulin sens. (index)^3^	6.9 ± 3.1	7.0 ± 3.8	7.1 ± 4.1	9.4 ± 5.2^ ∗∗a ∗∗∗b^
BG AUC (mg/dL)	597 ± 113	576 ± 116	604 ± 100	555 ± 88^∗∗∗b^
BG peak (mg/dL)	131 ± 23	127 ± 28	135 ± 28	126 ± 26^ ∗∗∗a ∗∗b^
Energy intake (Cal/d)	1855 ± 579	1649 ± 599	1756 ± 652	1271 ± 517^∗∗∗a ∗∗∗b^
Meals per day^4^	3.9 ± 0.7	3.9 ± 0.7	3.9 ± 0.6	3.7 ± 0.6^∗∗b^
Vegetable intake (g/d)	199 ± 209	227 ± 218	313 ± 242	424 ± 239^∗∗∗b^
Fruit intake (g/d)	183 ± 148	163 ± 153	221 ± 150	307 ± 259^∗a ∗∗b^

^1^Diary SD refers to BG SD of 21 measurements reported by each of 7 d diary.

^2^AUC: area under GTT curve.

^3^Whole body insulin sensitivity index [25].

^4^Meal was an event of higher energy intake than 20 kcal.

Values are expressed as mean ± SD. Peak values include different observations from those at 30' during GTT.* Asterisks* indicate significance (Student's *t*-test: *P < .05; **P < .01; ***P < .001) of longitudinal difference versus respective control group (a) or versus baseline values of the same group (b).

## List of Abbreviations

IHMP: Initial hunger meal pattern
AUC: Area under curve
BMI: Body mass index
BG: Blood glucose concentration
GTT: Oral glucose tolerance test
LBG: Low blood glucose
Diary-BG SD: Mean pre-meal blood glucose standard deviation reported by seven day diary
CRP: C reactive protein.
